# Theranostic Robot-Assisted Radical Prostatectomy: Things Understood and Not Understood

**DOI:** 10.3390/cancers15174288

**Published:** 2023-08-27

**Authors:** Chao-Yu Hsu, Che-Hsueh Yang, Min-Che Tung, Hung-Jen Liu, Yen-Chuan Ou

**Affiliations:** 1 Division of Urology, Department of Surgery, Tungs’ Taichung MetroHarbor Hospital, Taichung 435, Taiwan; t4361@ms.sltung.com.tw (C.-Y.H.); b101098093@tmu.edu.tw (C.-H.Y.); t1142@ms.sltung.com.tw (M.-C.T.); 2 Doctoral Program in Translational Medicine, National Chung Hsing University, Taichung 402, Taiwan; 3 Institute of Molecular Biology, National Chung Hsing University, Taichung 402, Taiwan; 4 The iEGG and Animal Biotechnology Center, National Chung Hsing University, Taichung 402, Taiwan; 5 Rong Hsing Translational Medicine Research Center, National Chung Hsing University, Taichung 402, Taiwan; 6 Department of Life Sciences, National Chung Hsing University, Taichung 402, Taiwan

**Keywords:** forecasting, humans, male, prostatic neoplasms, surgery, prostatectomy trends, robotic surgical procedures, robotic surgery history

## Abstract

**Simple Summary:**

Theranostic robot-assisted radical prostatectomy (T-RARP) means adopting RARP as an integration of diagnosis and therapy, and this concept has rarely been discussed. Before the advancement of imaging technologies, the diagnosis of prostate cancer (PCa) needed to rely on systematic biopsies, which had certain limitation of sensitivity and specificity. In our previous reports, even with preoperative benign biopsies, there were still large portions of incidental PCa in specimens of simple prostatectomies. We first introduced the feasibility of T-RARP in 2016 in patients with clinically highly suspected prostate cancer. In 2020, we developed a nomogram to identify the potential groups. With the advantages of imaging technologies, potential groups can be more easily identified, and the concept of T-RARP can be more widely adopted. However, no studies have discussed the benefits of T-RARP in these patients. Herein, we conducted this cohort study to explore the benefits of T-RARP for such patients and topics of T-RARP that are unclear and need to be unveiled in future studies.

**Abstract:**

Objective: This study aimed to explore the benefits of theranostic robot-assisted radical prostatectomy (T-RARP) for clinically highly suspicious prostate cancer (PCa) without proven biopsies. Material and Methods: Between February 2016 and December 2020, we included men with clinically highly suspicious PCa in this study. They were assessed to have possible localized PCa without any initial treatments, and were categorized into previous benign biopsies or without biopsies. Furthermore, another group of malignant biopsies with RARP in the same time frame was adopted as the control group. The endpoints were to compare the oncological outcome and functional outcome between malignant biopsies with RARP and T-RARP. *p* < 0.05 was considered to be significant. Results: We included 164 men with proven malignant biopsies treated with RARP as the control group. For T-RARP, we included 192 men. Among them, 129 were preoperatively benign biopsies, and 63 had no biopsies before T-RARP. Approximately 75% of men in the T-RARP group had malignant pathology in their final reports, and the other 25% had benign pathology. T-RARP provides several oncological advantages, such as a higher initial pathological T stage, lower Gleason grade, and lower odds of positive surgical margins. However, the biochemical recurrence rates were not significantly decreased. From our cohort, T-RARP (odds ratio with 95% confidence interval; erectile recovery: 3.19 (1.84–5.52), *p* < 0.001; continence recovery: 2.25 (1.46–3.48), *p* < 0.001) could result in better recovery of functional outcomes than malignant biopsies with RARP. Conclusions: For clinically highly suspicious PCa, T-RARP was able to detect around 75% of PCa cases and preserved their functional outcomes maximally. However, in 25% of men with benign pathology, approximately 6% would have incontinence and 10% would have erectile impairment. This part should be sufficiently informed of the potential groups considering T-RARP.

## 1. Introduction

According to the National Comprehensive Cancer Network (NCCN) guideline [1], localized prostate cancer (PCa) can be categorized into very low/low-, favorable/unfavorable intermediate-, and high/very high-risk groups. They are categorized according to various parameters such as biopsy results, prostate-specific antigen (PSA) levels, and image assessments of clinical stages [1]. In NCCN guidelines, radical prostatectomy (RP) is mainly reserved for men with localized PCa which could be completely excised and with life expectancy of >10 years [1]. 

Traditionally, RP is conducted following proof of malignancy, mostly from biopsies. This sequence of diagnostic biopsies and subsequent therapeutic extirpative surgeries had become the gold standard package to the majority of all solid organ tumors, including PCa. Although PCa is deemed a solid organ tumor, it relies on systematic biopsies to gain tissue proof of malignancy before targeted biopsy. These systematic biopsies were mostly performed using a transrectal ultrasound-guided (TRUS) method, whereas the targeted method incorporates 3T magnetic resonance imaging (MRI) with transrectal ultrasound. The ultrasound can be conducted using transrectal (TR) or transperineal (TP) methods. In the current literature, TR is similar to TP in terms of the detection rate in different target biopsies [2]. For men with suspected PCa, the clinically significant PCa detection rate of TRUS systematic biopsy may be similar to that of the targeted biopsy [3], which were 29.7% and 34.8%, respectively. The adverse effects of the targeted biopsy was 25% lower than that of the TRUS systematic biopsy. With the Clavien–Dindo system, incidences of grade 3 urinary tract infection and sepsis were lower in target biopsies [3]. 

In our experience, approximately 40% of men undergo preoperative benign biopsies of PCa in surgical specimens after simple prostatectomy [4]. Theranostics is an emerging trend in the modern medical industry that combines therapeutics and diagnostics. With advances in surgical devices, many surgeries, including robot-assisted RP (RARP), can be performed with them. In our previous experience [5], at one year after RARP, continence and potency were restored in approximately 98% and 86% of men, respectively. Considering this outstanding functional outcome, theranostic RARP (T-RARP) may be feasible. In men with highly suspicious PCa on 3T MRI, with a Prostate Imaging Reporting & Data System v2.0 (PI-RADS) score of 3 to 5 [6], the concept of T-RARP could avoid unnecessary surgeries, such as transurethral resection of the prostate (TURP). 

Currently, the concept of theranostics in PCa has mainly focused on nuclear medicine, based on molecular and tumor microenvironment aspects [7,8,9,10,11]. However, theranostic surgeries are rarely discussed. This study aimed to address our perspective on the role of T-RARP in terms of oncological and functional outcomes. 

## 2. Materials and Methods

### 2.1. Patients

Between February 2016 and December 2020, men visiting our clinic with highly suspected PCa who underwent 3T MRI or TRUS-guided biopsy were screened for eligibility to be enrolled. The inclusion criteria were localized PCa without previous androgen deprivation therapy (ADT) or other treatments, such as radiotherapy or brachytherapy. PI-RADS scoring was interpreted according to our protocol [12,13,14] by our radiologists, and the results were subjected to peer review. During shared decision making with men with PI-RADS scoring ≥3, they would be offered MRI–TRUS fusion-guided biopsy via TP or TRUS-guided biopsy via TR. If they refused to undergo biopsies, the treatment options were further discussed with the project administration (institutional review board approval No. R109036) and the purpose of T-RARP was explained for at least 30 min. An informed consent sheet would be signed by the patients. For men with malignant biopsies, feasible therapeutic choices would be suggested according to their risk categories in the latest NCCN guideline after diagnosis. 

Men without previous 3T MRI would undergo 1.5T MRI examination after malignant pathological results from TRUS-guided biopsy. After confirming localized PCa on images, they would be suggested feasible therapeutic choices according to their risk categories in the latest NCCN guidelines after diagnosis. Men with benign pathological results are recommended to undergo PSA and digital rectal examination (DRE) every 6 months. For men who select active surveillance (AS), confirmatory biopsies should be suggested at 2 years after diagnostic biopsies. For men with benign pathological results but highly suspected PCa according to their clinical signs, such as high PSA or fast PSA velocity, T-RARP should be introduced by the project administration. After understanding and agreeing with the contents of T-RARP, they signed an informed consent form before surgery. 

The exclusion criteria included metastatic PCa, previous ADT, previous definite therapies other than RP, incomplete medical records, follow-up of <2 years, men with AS but without confirmatory biopsies, and men with preoperative incontinence and erectile dysfunction. Personal and medical histories were extracted from medical charts and nursing records. 

### 2.2. Oncological Outcome and Functional Outcomes 

Regarding oncological outcomes, after T-RARP and RARP, men would have their PSA followed up every 3 months in the first 2 years, and then every 6 months for the next 2 years. An undetectable PSA level was defined as 0.008 ng/mL. Biochemical recurrence (BCR) was defined to be a PSA concentration of 0.2 ng/mL at 4 weeks after the first test [15]. 

For functional outcomes, erectile function (EF) and continence were the two parameters assessed. Men who underwent RARP or T-RARP were further investigated when their EF and continence were restored. EF was assessed based on the International Index of Erectile Function-5 (IIEF-5) questionnaire, and EF dysfunction was defined as IIEF-5 scores < 21. Continence was assessed based on the pads used and was defined to be 0–1 pad/day. Postoperative follow-ups were arranged every 3 months during the first 2 years, and then every 6 months for the next 2 years.

### 2.3. Statistical Methods 

After checking the skewness and kurtosis of the data, nonparametric and parametric analytical methods were applied. The dichotomous outcome of BCR was used as the independent variable, and significant dependent variables in the univariate regression analysis were further merged into the multivariate regression analysis. A *p*-value of <5% was considered significant. The analysis was performed using the R software (version 4.0.5, R Core Team (2021); R: language and environment for statistical computing; R Foundation for Statistical Computing, Vienna, Austria. URL: https://www.R-project.org/, accessed on 3 April 2021) and SPSS (Version 25, IBM Corp., Armonk, NY, USA).

## 3. Results

### 3.1. General Outcomes

**Figure 1:**
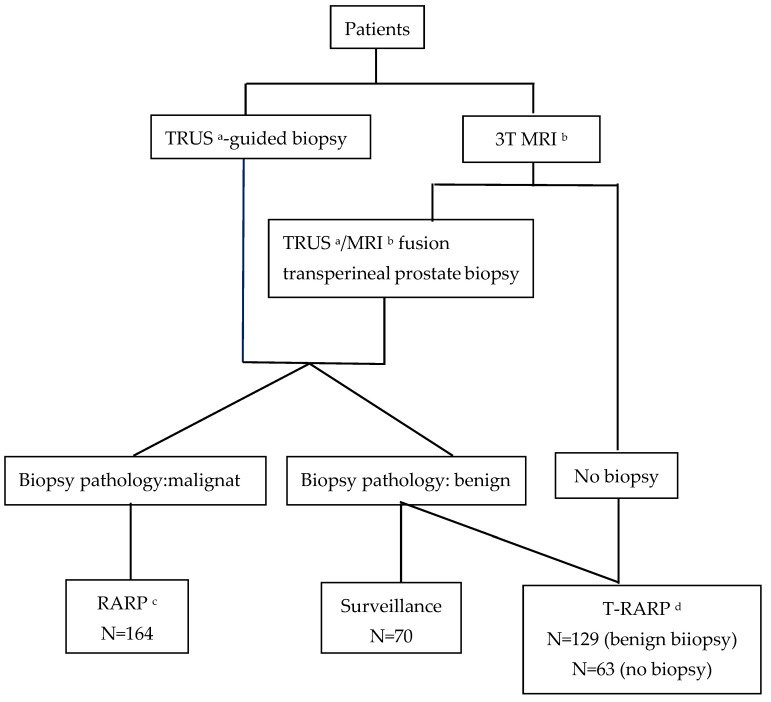
Flowchart of this study. ^a^ transrectal ultrasound; ^b^ magnetic resonance imaging; ^c^ robot-assisted radical prostatectomy; ^d^ theranostic robot-assisted radical prostatectomy.

After obtaining informed consent, 426 men agreed to participate. Of 78 men, 8 (10%) in surveillance selected RARP after malignant confirmatory biopsies, and the remaining 70 men stayed on surveillance, instead of selecting RARP or T-RARP (Figure 1). The mean follow-up was 30.52 ± 8.02 months. The data of 356 men who underwent either T-RARP or RARP are listed in Table 1. 

There were 29 (23%) men in Group 2 with benign pathological results. In the remaining 100 men with PCa, their median age, body mass index (BMI), and PSA was 65 (inter-quartile range [IQR]:8) (non-PCa: 61 [8]; *p* < 0.001, Mann–Whitney U test) years old, 24.93 (IQR: 3.51) (non-PCa: 25.1 [3.87]; *p* = 0.892, Mann–Whitney U test) kg/m^2^, and 9.60 (IQR: 4.09) (non-PCa: 7.14 [3.66]; *p* = 0.001, Mann–Whitney U test) ng/mL, respectively. In Group 2, fifty-nine (59%) men with PCa had a preoperative abnormal digital rectal exam (DRE), while only seven (24.1%) men without PCa had it (*p* = 0.001, Fisher’s exact test). In men with PCa in Group 2, 31 (31%) selected fusion biopsy and 9 (31%) selected non-PCa (*p* = 1, Fisher’s exact test). In preoperative biopsies, no significant differences in total cores (*p* = 0.620, Mann–Whitney U test), percentage of positive cores (*p* = 0.97, Mann–Whitney U test), and resected prostate weight (*p* = 0.551, Mann–Whitney U test) were observed between the PCa and non-PCa groups in Group 2. A total of 53 men underwent 3T-MRI with PI-RADS readings. Fifteen of twenty-five (60%) men with a PI-RADS score of 3 had PCa. Regarding a PI-RADS score of 4, 14 of 19 (74%) men had PCa. In the PI-RADS score 5, eight of nine men had PCa (89%). Categorized by a PI-RADS score of 3, a PI-RADS score of 4/5 was not associated with an increased risk of PCa (univariate logit model, odds ratio (OR) with 95% confidence interval (CI): 2.43 (0.73–8.33). The median PSA in PI-RADS score 4/5 was 9.49 (IQR: 4.43) ng/mL, and it was 9.12 (IQR: 5.48) ng/mL in men with PI-RADS score 3 (*p* = 0.748, Mann–Whitney U test). The median age of men with PI-RADS 4/5 was 64 (IQR: 10) years and that of men with a PI-RADS score of 3 was 63 (IQR: 8) years (*p* = 0.242, Mann–Whitney U test). Abnormal DRE findings were not related to the odds categorized as a PI-RADS score 4/5 (OR with 95% CI: 0.58 (0.19–1.72), *p* = 0.325). In the multivariable logit model of having PCa, age (OR with 95% CI: 1.22(1.09–1.35), *p* < 0.001), PSA (OR with 95% CI: 1.24(1.04–1.48), *p* = 0.014), and abnormal DRE (OR with 95% CI: 3.84 (1.38–10.64), *p* = 0.01) were all related to the cancerous outcome. Among men with benign pathology, two (6.8%) had incontinence and two (6.8%) had erectile dysfunction. 

In Group 3, 46 (73%) men had cancer in their final pathological reports. In men having PCa in group 3, their median age, BMI, and PSA was 61 (IQR: 6) (non-PCa: 61 (IQR: 8); *p* = 0.254, Mann–Whitney U test) years old, 24.11 (IQR: 4.90) (non-PCa: 24.74 (IQR: 6.49); *p* = 0.739, Mann–Whitney U test) kg/m^2^, and 10.12 (IQR: 3.44) (non-PCa: 7.1 (IQR: 5.4); *p* = 0.007, Mann–Whitney U test) ng/mL, respectively. In Group 3, 29 (63%) men demonstrated cancerous pathology and abnormal preoperative DRE findings, whereas only five (29%) men without PCa had abnormal DRE findings (*p* = 0.024, Fisher’s exact test). The median resected prostate weight was 49 g (IQR: 20) for men with PCa in Group 3 and 42 g (IQR: 25) for non-PCa men in Group 3 (*p* = 0.177, Mann–Whitney U test). In men with PCa in Group 3, 11 (23.9%), 22 (47.8%), and 13 (28.3%) patients had PI-RADS scores of 3, 4, and 5, respectively. Among the non-PCa men in Group 3, nine (52.9%) and eight (47.1%) had PI-RADS scores of 3 and 4, respectively. Between these two subgroups in Group 3, the proportion of patients with a PI-RADS score of 3 was significantly higher in men without PCa (*p* = 0.037, Fisher’s exact test). In the multivariable logit model with a dependent variable of cancerous outcome, PSA (OR with 95% CI: 1.33 (1.07–1.67), Wald: 6.42) was the only significant variable after controlling PI-RADS scores (OR with 95% CI: 0.09–1.4, reference: PI-RADS score = 3) and DRE findings (OR with 95% CI: 0.08–1.20, reference: normal DRE finding). In addition, elevated PSA levels did not increase the odds of having a PI-RADS score of 4/5 (OR with 95% CI: 1.02 (0.86–1.21), *p* = 0.797). However, abnormal DRE findings were related to the less odds of having a PI-RADS score of 3 (OR with 95% CI: 0.23 (0.07–0.72), *p* = 0.012). Thus, we re-examined the multivariable logit model with PSA/DRE and PSA/PI-RADS scores as independent variables. In PSA/DRE, both of them were significantly related to the cancerous yield (PSA: OR with 95%CI = 1.33 (1.07–1.66), *p* = 0.011; DRE (normal DRE with reference to the abnormal DRE): OR with 95% CI = 0.23 (0.07–0.85) *p* = 0.027). In PSA/PI-RADS scores, both of them were remarkably related to the cancerous yield (PSA: OR with 95% CI = 1.33 (1.07–1.66), *p* = 0.011; PI-RADS scores (score 3 with reference to score 4/5): OR with 95%CI = 0.25 (0.07–0.92), *p* = 0.036). In men with benign pathology, incontinence occurred in one patient (5.8%) and erectile dysfunction occurred in two (11.7%). 

In adverse effects, urine leak occurred in 1% in each group (Group 1: two men; Group 2: one man; Group 3: one man). Lymphocele occurred in 2% in Group 1 (three men), and 1% in Group 2 (one man). Bladder neck contracture (BNC) occurred 1% in Group 1 (one man; time-to-BNC: 27 months). For lymphocele, we arranged external drainage placed by radiologists. For BNC, we dealt with it by endoscopic incision in the first place, and urethral dilation if symptoms recurred.

### 3.2. Oncological Outcomes 

**Figure 2:**
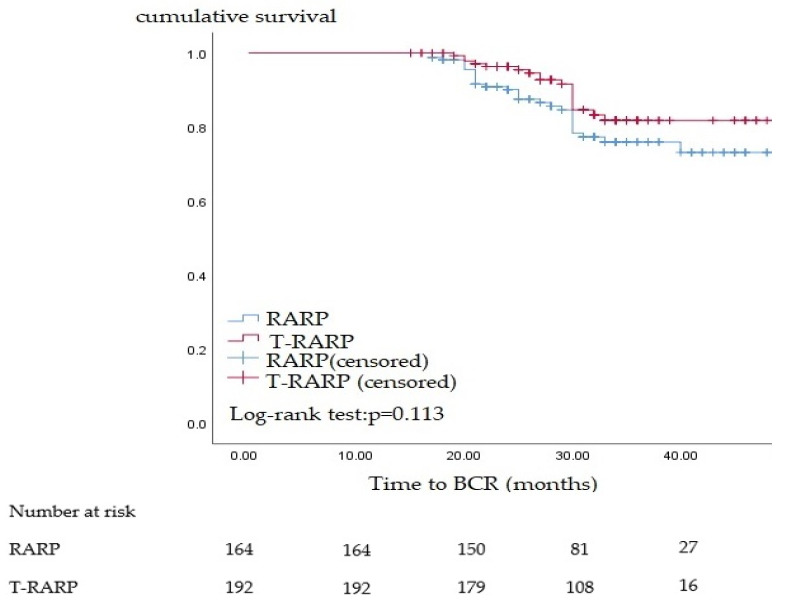
Survival analysis of biochemical recurrence (BCR) by categorization of robot-assisted radical prostatectomy (RARP) and theranostic robot-assisted radical prostatectomy (T-RARP).

During the follow-up, the total BCR rates were 13.6% and 18.3% in men after T-RARP and RARP, respectively. As presented in Table 2, we tested various independent parameters to predict the BCR. However, we failed to identify sufficient independent parameters to establish multivariate analysis. In addition, neither T-RARP nor RARP was associated with BCR (Table 2 and Figure 2). 

### 3.3. Functional Outcomes 

In the outcome of the recovery of erectile function (Table 3), the most significant factor in the multivariable logit regression was the operative method (Wald: 17.10), followed by the use of a vacuum device (Wald: 13.75) and bilateral nerve sparing during the operation (Wald: 10.68). Regarding continence recovery (Table 4), the most significant factor affecting continence recovery was the operative method (Wald: 13.42), followed by pelvic muscle training (Wald: 8.02) and personal history of drinking (Wald: 4.29). 

## 4. Discussion

In this cohort, 58% men with clinically highly suspicious PCa after T-RARP would be categorized into intermediate risk (IR) localized PCa at least. These type of patients should benefit the most from T-RARP, since active surveillance was controversial in them [16]. Although there were still 42% men belonging to grade group 1, many factors would still affect the choice of active surveillance such as patients’ compliance and lack of evidence supporting the selection criteria and surveillance protocols [17]. In a large cohort study, 36% men would change to definite therapies after 5-year active surveillance [18]. Although the T-RARP would not contribute to better cancer-specific survival in them, it would lower their treatment shift in the first 5 years. Additionally, the enrolled patients of T-RARP groups in our cohort seemed to be younger than the previous large-scale active surveillance cohort [18]. This way, the treatment shift of our cohort would possibly be higher if they selected active surveillance in the first place.

In a previous study, we were the first to address the concept of T-RARP [19] and to build a normogram based on it to predict PCa in specimens [20]. However, the benefits of T-RARP were not discussed in our previous work. In this study, we discuss T-RARP based on two aspects of radical prostate surgery: oncological and functional outcomes. Predicting malignancy preoperatively by using T-RARP is challenging [20]. Based on our previous works [4,20] and the present one, the application of PSA to predict malignancy in either preoperative benign or no biopsies was robust. Interestingly, we observed different roles of PI-RADS scores in patients with and without preoperative biopsies. Using PI-RADS scores to predict real malignancy would be feasible in men without preoperative biopsies, and its predictive role might overlap with that of DRE. This could be attributed to the fact that some 3T MRI images were obtained after systematic TRUS-guided biopsies. The chronic inflammation caused by the systematic biopsies might lead to the false-positive results under such conditions [21]. Thus, the best experience from our cohort suggests that PI-RADS scores used to predict malignancy from T-RARP should be performed before biopsies. However, a limitation of analyzing the predictive factors was that the sample size in Group 3 was small. This is reflected in some evident type-2 errors observed, such as age. In addition, the overall number of independent variables used to establish the multivariable logit model was less than required. Thus, the topics of the T-RARP need to be expanded to explore topics that are unclear. 

In our experience, approximately 19% of men with incidental PCa undergo surgery for benign prostate enlargement, which greatly affects their quality of life [4]. In this cohort, 77% of men with benign preoperative biopsies but considered at a high risk of harboring PCa developed malignancy after T-RARP, and 73% of men considered at a high risk of harboring PCa but without biopsies developed PCa after T-RARP. For incidental PCa, although it was mainly detected in the low pathological T stage or Gleason grade groups, deferred RP in these patients might still increase the risk of PCa upgrade [22], and this risk remained the same in men with potential cancer but with deferred therapies because of the inability to detect PCa. Within this cohort, 10% of the men opted for active monitoring and underwent malignant confirmatory biopsies. From our T-RARP data, a certain proportion of men were categorized into the favorable intermediate-risk group. Especially for patients with T2b PCa, >90% would prefer deferred RP after initial AS [23].

Judging from the men after T-RARP, those with preoperative benign biopsies or those without preoperative biopsies tended to have lower preoperative PSA, lower Gleason grade, and pathological T stage. These factors make it difficult for urologists to perform biopsies in current clinical practice. Thus, the main reason that T-RARP should be introduced to patients is that it can provide the purpose of diagnosis and therapy simultaneously. In addition, T-RARP has several advantages, such as lower rates of positive surgical margins. However, despite the several advantages in the oncological outcomes provided by T-RARP, no remarkable difference in the BCR OR was observed between these two operative methods. We postulate that this type-2 error resulted from the general conditions of our data. First, our follow-up period was too short to observe a difference in the BCR. For example, for organ-confined PCa with positive surgical margins but without extracapsular extension, the median time from the PSA nadir to BCR might be 4 years [24]. Thus, BCR in the potential adverse risk groups might not have occurred during our observation period. This type-2 error was also evident when a logit regression model for BCR was established. Several factors affecting the outcome of BCR were not effective in our cohort [25,26,27,28], especially PSA level [26]. 

The most beneficial effect of T-RARP over RARP was functional outcome. In our assessment model, T-RARP was the most effective factor in preserving erectile and continence functions and was even more effective than postoperative rehabilitation such as pelvic muscle training, use of a vacuum device, and medication. This may be the result of men selecting T-RARP having more organ-confined PCa than men with malignant biopsies and RARP. In this way, techniques of nerve sparing, such as interfascial or intrafascial dissection [29], could be maximally performed, and the urethral stump could be preserved as long as possible [30]. However, some adverse indicators, such as underlying diabetes mellitus, may affect the recovery of functional outcomes. Identifying such factors could help urologists avoid the side effects of T-RARP as much as possible, especially when approximately 25% of men experience benign results after T-RARP. Functional outcomes after T-RARP may be key factors that influence patient decision making. Informed consent for this part of the study was essential. For men deciding active monitoring on PCa, the natural decaying process would increase impaired EF by approximately 20% after 6 years, and the lower urinary tracts symptoms by approximately 20% as well [31]. The effects of RARP on functional outcomes are immediate and dramatic. In our cohort, approximately 6% of men with benign pathology after T-RARP had incontinence and 10% of men had erectile dysfunction. These situations must be sufficiently explained before conducting T-RARP. 

We were the first to propose the application of RARP in theranostics [19,20], and this cohort study was the first to discuss T-RARP based on oncological and functional outcomes. This study demonstrated that T-RARP could maximally preserve functional outcomes compared to malignant biopsies with RARP. However, this is a double-edged sword as 25% men still have a benign pathology after T-RARP, and a small portion of them were at risk of impaired EF (10%) and incontinence (6%). Those who consider T-RARP should be sufficiently informed regarding this. In addition, identifying potential factors that negatively affect functional outcomes is essential for urologists when explaining and suggesting T-RARP. Currently, limited information is available for this study. Oncological outcomes were not understood in this study. Limited by several evident type-2 errors, although T-RARP has several pathological advantages for BCR, we could not draw such a conclusion because of statistical problems. This unresolved topic requires larger cohorts with long-term surveillance in the future. 

## 5. Conclusions

For clinically highly suspicious PCa, T-RARP was able to detect around 75% of PCa cases. For 75% of men, T-RARP could maximally preserve functional outcomes, although its superiority on oncological outcomes over malignant biopsies with RARP remains unconfirmed. Informed consent was still important, as approximately 25% of the men had a benign pathology in their final reports. Among them, approximately 6% of men had incontinence and 10% of men had impaired EF. Avoiding such side effects is important, and preoperative indicators should be explored. 

## Figures and Tables

**Table 1:** General data in each group. † Men with benign pathological results were excluded from analysis. ⁋ Student’s t test. ‡ Chi-square test with Yates’ correction. § Mann–Whitney U test.

	Group 1: Malignant Biopsies with RARP ^a^ N = 164	Group 2: Benign Biopsies with T-RARP ^b^ N = 129	Group 3: No Biopsies with T-RARP ^b^ N = 63	*p*-Value
Age (years old; mean ± SD ^d^)	66.01 ± 3.84	63.76 ± 5.43	60.95 ± 4.72	Group 1 vs. group 2: *p* < 0.001 ⁋ Group 1 vs. group 3: *p* < 0.001 ⁋ Group 2 vs. group 3: *p* < 0.001§
BMI ^c^ (Kg/m^2^; mean ± SD ^d^)	24.83 ± 3.02	24.94 ± 2.94	24.55 ± 3.54	Group 1 vs. group 2: *p* = 0.631 ⁋ Group 1 vs. group 3: *p* = 0.486 ⁋ Group 2 vs. group 3: *p* = 0.281 §
PSA (ng/mL; mean ± SD ^d^)	12.17 ± 4.74	9.82 ± 3.83	9.45 ± 3.19	Group 1 vs. group 2: *p* < 0.001 ⁋ Group 1 vs. group 3: *p* < 0.001 ⁋ Group 2 vs. group 3: *p* = 0.885 §
DRE (n; %)				Group 1 vs. group 2: *p* = 0.440 ‡ Group 1 vs. group 3: *p* = 0.685 ‡ Group 2 vs. group 3: *p* = 0.838 ‡
Normal	72 (43.9%)	63 (48.8%)	29 (46%)	
Abnormal	92 (56.1%)	66 (51.2%)	34 (54%)	
Biopsy methods (n; %)				-
TRUS-guided biopsy	128 (78%)	89 (69%)	-	
MRI/TRUS-guided fusion biopsy	36 (22%)	40 (31%)	-	
No biopsy	-	-	63 (100%)	
Resected prostate weight (gram; mean ± SD ^d^)	49.48 ± 14.42	48.22 ± 15.19	48.11 ± 14.29	Group 1 vs. group 2: *p* = 0.331 ⁋ Group 1 vs. group 3: *p* = 0.575 ⁋ Group 2 vs. group 3: *p* = 0.913 §
Cancer percentage (%; mean ± SD ^d^)	19.21 ± 9.60	† 20.26 ± 12.23	† 16.67 ± 10.45	Group 1 vs. group 2: *p* = 0.819 ⁋ Group 1 vs. group 3: *p* = 0.474 ⁋ Group 2 vs. group 3: *p* = 0.117 §
Pathological Gleason Grade Group (n; %)		†	†	Group 1 vs. group 2: *p* < 0.001 ‡ Group 1 vs. group 3: *p* < 0.001 ‡ Group 2 vs. group 3: *p* = 0.626 ‡
1	10 (6.1%)	54 (54%)	26 (56.5%)	
2	32 (19.5%)	28 (28%)	13 (28.3%)	
3	76 (46.3%)	13 (13%)	5 (10.9%)	
≥4	46 (28%)	5 (5%)	2 (4.3%)	
Gleason group upgrading compared with the biopsy		†		-
Yes	134 (81.7%)	-	-	
No	30 (18.3%)	-	-	
Percentage of positive lymph nodes (%; mean ± SD ^d^)	42.78 ± 20.87	† 15.43 ± 12.45	† 11.06 ± 9.63	Group 1 vs. group 2: *p* < 0.001 ⁋ Group 1 vs. group 3: *p* < 0.001 ⁋ Group 2 vs. group 3: *p* < 0.001 §
Pathological T stage (n; %)		†	†	Group 1 vs. group 2: *p* < 0.001 ‡ Group 1 vs. group 3: *p* < 0.001 ‡ Group 2 vs. group 3: *p* = 0.596 ‡
2a	31 (18.9%)	47 (47%)	26 (56.5%)	
2b	8 (4.9%)	26 (26%)	7 (15.2%)	
2c	38 (23.2%)	13 (13%)	8 (17.4%)	
3a	49 (29.9%)	9 (9%)	2 (4.3%)	
3b	38 (23.2%)	5 (5%)	3 (6.5%)	
Positive surgical margins (n; %)		†	†	Group 1 vs. group 2: *p* < 0.001 ‡ Group 1 vs. group 3: *p* < 0.001 ‡ Group 2 vs. group 3: *p* = 0.415 ‡
Yes	62 (37.8%)	25 (25%)	9 (19.5%)	
No	102 (62.2%)	75 (75%)	37 (80.5%)	
Incontinence (n)				Group 1 vs. group 2: *p* = 0.004 ‡ Group 1 vs. group 3: *p* = 0.003 ‡ Group 2 vs. group 3: *p* = 0.465 ‡
Yes	96 (58.5%)	54 (41.9%)	23 (36.5%)	
No	68 (41.5%)	75 (58.1%)	40 (63.5%)	
ED ^e^ (n)				Group 1 vs. group 2: *p* < 0.001 ‡ Group 1 vs. group 3: *p* = 0.009 ‡ Group 2 vs. group 3: *p* = 0.744 ‡
Yes	88 (53.7%)	42 (32.6%)	24 (38.1%)	
No	76 (46.3%)	87 (67.4%)	39 (61.9%)	
BCR ^f^ (n)		†	†	Group 1 vs. group 2: *p* = 0.018 ‡ Group 1 vs. group 3: *p* = 0.841 ‡ Group 2 vs. group 3: *p* = 0.235 ‡
Yes	30 (18.3%)	11 (11%)	9 (19.5%)	
No	134 (81.7%)	89 (89%)	37 (80.5%)	

^a^ Robot-assisted radical prostatectomy; ^b^ Theranostic robot-assisted radical prostatectomy; ^c^ Body mass index; ^d^ Standard deviation; ^e^ Erectile dysfunction; ^f^ Biochemical recurrence.

**Table 2:** Relationship of biochemical recurrence (BCR) oncological outcome with each parameter.

	Univariable Logit Regression (OR with 95% CI)	*p*-Value
Age (years old)	0.99 (0.93–1.06)	0.830
PSA (ng/mL)	1.02 (0.96–1.09)	0.498
DRE		
Abnormal	Reference	
Normal	0.72 (0.38–1.37)	0.321
Pathological Gleason Grade Group		
1	Reference	
2	1.34 (0.15–12.00)	0.792
3	3.65 (0.45–29.47)	0.225
≥4	4.82 (0.56–41.77)	0.153
Pathological T stage		
2a	Reference	
2b	0.62 (0.24–1.64)	0.334
2c	0.98 (0.32–2.98)	0.969
3a	1.34 (0.50–3.58)	0.556
3b	0.73 (0.25–2.12)	0.564
Percentage of positive lymph nodes	1.01 (0.99–1.02)	0.357
Resected prostate weight	1.02 (1.00–1.04)	0.052
Cancer percentage	1.02 (0.99–1.04)	0.272
Positive surgical margins		
No	Reference	
Yes	0.71 (0.38–1.31)	0.274
Operative methods		
T-RARP	Reference	
Malignancy biopsies and RARP	1.59 (0.85–2.99)	0.150

**Table 3:** Factors affecting the recovery of erectile function.

	Univariable Logit Regression (OR with 95% CI)	*p*-Value	Multivariable Logit Regression (OR with 95% CI)	*p*-Value
Age (years old)	1.06 (1.02–1.11)	0.007	1.02 (0.97–1.07)	0.457
Drinking				
Yes	Reference		Reference	
No	0.50 (0.32–0.76)	0.001	0.71 (0.44–1.15)	0.168
Bilateral nerve sparing				
Yes	Reference		Reference	
No	0.37 (0.23–0.59)	<0.001	0.43 (0.26–0.72)	0.001
Diabetes mellitus				
Yes	Reference		Reference	
No	0.52 (0.34–0.79)	0.002	0.63 (0.39–0.99)	0.050
Pelvic floor muscle training				
No	Reference			
Yes	1.29 (0.81–2.06)	0.288		
Use of tadalafil 5 mg daily				
Yes	Reference		Reference	
No	0.55 (0.35–0.86)	0.008	0.55 (0.33–0.90)	0.017
Use of vaccum device				
Yes	Reference		Reference	
No	0.53 (0.31–0.90)	0.018	0.30 (0.16–0.57)	<0.001
Operative methods				
Malignant biopsies and RARP	Reference		Reference	
T-RARP	2.37 (1.54–3.65)	<0.001	3.19 (1.84–5.52)	<0.001

**Table 4:** Factors affecting the recovery of continence.

	Univariable Logit Regression (OR with 95% CI)	*p*-Value	Multivariable Logit Regression	*p*-Value
Age (years old)	0.98 (0.94–1.02)	0.273		
Drinking				
Yes	Reference		Reference	
No	1.61 (1.04–2.48)	0.033	1.61 (1.03–2.51)	0.038
Bilateral nerve sparing				
Yes	Reference			
No	0.82 (0.53–1.27)	0.378		
Diabetes mellitus				
Yes	Reference			
No	0.76 (0.50–1.15)	0.198		
Pelvic floor muscle training				
No	Reference		Reference	
Yes	1.92 (1.19–3.10)	0.007	2.04 (1.25–3.34)	0.005
Operative methods				
Malignant biopsies and RARP	Reference		Reference	
T-RARP	2.16 (1.41–3.29)	<0.001	2.25 (1.46–3.48)	<0.001

## Data Availability

The data can be shared up on request.
